# BOADICEA model: updates to the *BRCA2* breast cancer risks for ages 60 years and older

**DOI:** 10.1038/s44276-024-00079-1

**Published:** 2024-07-24

**Authors:** Lorenzo Ficorella, Xin Yang, Douglas F. Easton, Antonis C. Antoniou

**Affiliations:** 1 Centre for Cancer Genetic Epidemiology, Department of Public Health and Primary Care, University of Cambridge, Cambridge, UK; 2 Centre for Cancer Genetic Epidemiology, Department of Oncology, University of Cambridge, Cambridge, UK

## Abstract

Breast cancer risks in older *BRCA2* pathogenic variant carriers are understudied. Recent studies show a marked decline in the relative risk at older ages. We used data from two large studies to update the breast cancer risks in the BOADICEA model for *BRCA2* carriers 60 years and older.

The Breast and Ovarian Analysis of Disease Incidence and Carrier Estimation Algorithm (BOADICEA), implemented in the CanRisk tool (www.canrisk.org) [1], can be used to predict the risk of developing breast cancer (BC) and the likelihood of carrying pathogenic variants in breast cancer susceptibility genes [2, 3]. The model combines the effects of rare genetic variants, polygenic risk scores, cancer family history, mammographic density and several other lifestyle/hormonal risk factors.

The risks associated with *BRCA1* and *BRCA2* pathogenic variants (PVs) used in BOADICEA were originally estimated using complex segregation analysis in a large series of families carrying PVs. The model estimated relative risks (RRs) of breast and ovarian cancer up to age 70 years, as there were few data at older ages [4]. The model assumed breast cancer log-relative risks that are piecewise linear functions of age: in particular, the RRs were estimated to increase from 8.99 at age 59 to 13.1 at age 69 years. To implement the model for older individuals, the RR was assumed to remain constant at 13.1 for ages 70 years and older [2, 4]. However, more recent data [5–7] suggest that the BC RRs decrease with age, and that the RR for ages 70 and over is much lower; therefore, the original RR estimate of 13.1 is most likely an overestimate for older *BRCA2* carriers.

We therefore updated the RRs in BOADICEA by re-deriving the piecewise log-RR linear function for ages 60 and over. We first estimated a revised RR for ages 70–79 using results from two recent large studies [5, 6]. The first, BRIDGES, is a case–control analysis based on panel testing in ~50,000 breast cancer cases and 50,000 controls from population-based studies; this study showed a clear decline in the odds ratio (OR) by age and reported an OR of $3.05$ [95% CI: 2.14–4.35] at ages 60–80 ([5], Table S12). The second study is a prospective cohort study that included 1610 *BRCA2* PV carriers unaffected at baseline; this study reported a RR for ages 71–80 of 6$.6$ [95% CI: 3.0–14.7]. A third large case–control also found a decline with age and reported (graphically only) an OR ~3 at ages 70–80 [7]. We derived a meta-analysis RR estimate for ages 70 and over (3.38) as the inverse-variance weighted average of the two available estimates (using the reported sample sizes and 95% confidence intervals to compute the variances). To ensure that relative risks decrease smoothly with age, a piecewise log-linear function with age was then fitted such that the BC RR decreases from 9.05 at age 60 (as in the previous model) to 3.38 at ages 70, and constant thereafter. Note that the RRs here correspond to the average RR for *BRCA2* carriers relative to the population incidences, averaged over all polygenic effects, which are the inputs to the model [2]. The residual polygenic component in the model was adjusted to account for the change in the RRs and to ensure that the model remains internally consistent for predicting the breast cancer familial relative risks [3].

Figure 1 shows the revised age-dependent BC RRs and incidences for *BRCA2* PV carriers (Fig. 1a, b), and the predicted cumulative risk of developing BC for *BRCA2* PV carriers (Fig. 1c, d). Based on the revised RRs, the predicted cumulative risk of developing BC for *BRCA2* PV carriers with unknown family history (Fig. 1c) remains identical to the previous BOADICEA model until around age 61, and increases more slowly thereafter, reaching 58% by age 80. This compares to the 77% BC risk by age 80 obtained in the previous version. For a *BRCA2* PV carrier with an affected mother at age 40 (Fig. 1d), the breast cancer risk by age 80 is predicted to be 72.5%, compared to the 87% BC risk obtained in the previous version. These revised predictions are more consistent with both the population-based penetrance estimates (e.g. ~43% in the BRIDGES study [5], ~50% reported by Hu et al. [7]) and estimates from carriers with cancer family history (72% [95% CI: 65–79%] reported by Kuchenbaecker et al. [6]).

**Fig. 1:**
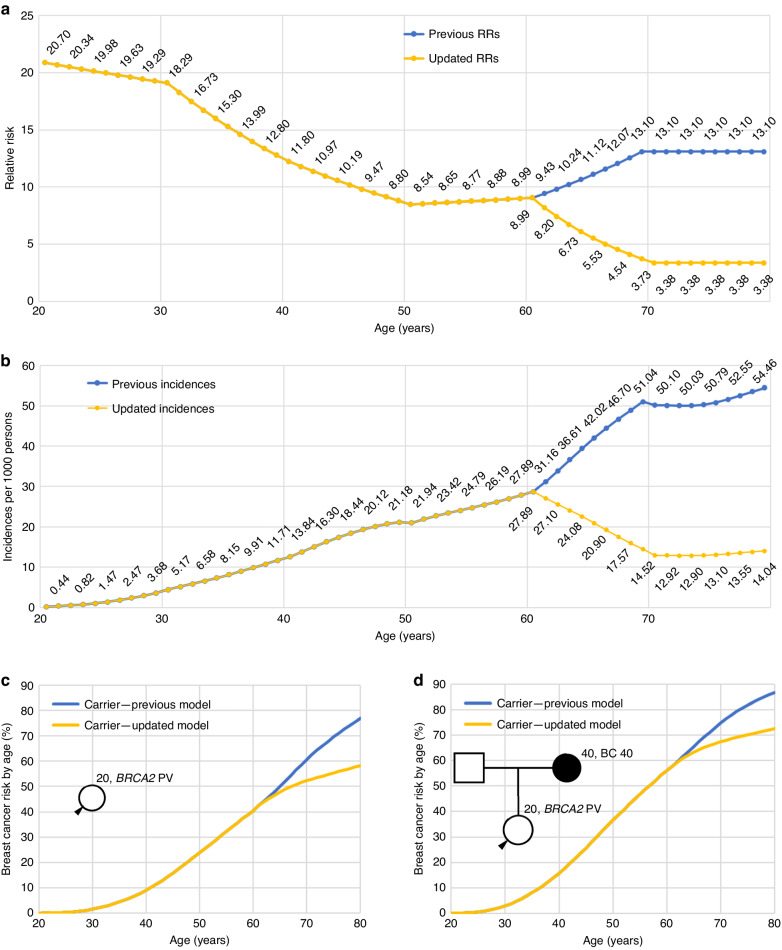
Comparison between previous and updated BOADICEA parameters, and between the corresponding predicted BC risks. **a** Previous and updated *BRCA2* relative risks in BOADICEA/CanRisk v2.4. **b** Previous and updated BC incidences for *BRCA2* mutation carriers (UK cohort born in 2000). **c** Revised cumulative breast cancer risk for the average *BRCA2* pathogenic variant (PV) carrier (20 years old, born in 2000) using previous and updated *BRCA2* RRs. **d** Corresponding cumulative risks for *BRCA2* PV carrier (20 years old, born in 2000) with an affected mother (40 years old, born in 1980) diagnosed at age 40.

We note that the data are still limited in this age group that there is still uncertainty in the risk estimates, and that better estimates may be become available with further data. The estimates from the cohort studies are likely to be upwardly biased (as estimates of the RRs relative to the general population) as the cohort is oversampled for carriers with a family history. However, the BRIDGES study (though large) has relatively few control carriers, and we did not want to rely solely on the results from one study. All studies, however, show a consistent decline in the RR with age; this is also more consistent with the pattern seen for most cancer susceptibility genes (e.g. *MSH2* for colorectal and endometrial cancers [8]). The new estimates are therefore likely to be more realistic. A recent validation study in *BRCA1* and *BRCA2* PV carriers suggests that this model provides well-calibrated risks in both *BRCA2* PV carriers under and over the age of 50 years [9].

Note that we have now also updated the corresponding ovarian cancer risk model to include these new breast cancer RRs [10] (the ovarian model is affected by this change as it also considers breast cancer family history). The updated breast and ovarian cancer models are available through the CanRisk v2.4 tool.

## Preprint

A previous version of this manuscript was published as a preprint [11].

## Data Availability

Data sharing not applicable to this article as no datasets were generated or analysed during the current study.

## References

[1] Carver T, Hartley S, Lee A, Cunningham AP, Archer S, Babb de Villiers C, et al. CanRisk Tool—a web interface for the prediction of breast and ovarian cancer risk and the likelihood of carrying genetic pathogenic variants. Cancer Epidemiol Biomarkers Prev. 2021;30:469–73. 10.1158/1055-9965.EPI-20-1319. 33335023 10.1158/1055-9965.EPI-20-1319 PMC7611188

[2] Lee A, Mavaddat N, Cunningham A, Carver T, Ficorella L, Archer S, et al. Enhancing the BOADICEA cancer risk prediction model to incorporate new data on RAD51C, RAD51D, BARD1 updates to tumour pathology and cancer incidence. J Med Genet. 2022;59:1206–18. 10.1136/jmedgenet-2022-108471. 36162851 10.1136/jmedgenet-2022-108471 PMC9691826

[3] Lee A, Mavaddat N, Wilcox AN, Cunningham AP, Carver T, Hartley S, et al. BOADICEA: a comprehensive breast cancer risk prediction model incorporating genetic and nongenetic risk factors. Genet Med. 2019;21:1708–18. 10.1038/s41436-018-0406-9. 30643217 10.1038/s41436-018-0406-9 PMC6687499

[4] Antoniou AC, Cunningham AP, Peto J, Evans DG, Lalloo F, Narod SA, et al. The BOADICEA model of genetic susceptibility to breast and ovarian cancers: updates and extensions. Br J Cancer. 2008;98:1457–66. 10.1038/sj.bjc.6604305. 18349832 10.1038/sj.bjc.6604305 PMC2361716

[5] Breast Cancer Association Consortium, Dorling L, Carvalho S, Allen J, Gonzalez-Neira A, Luccarini C, et al. Breast cancer risk genes—association analysis in more than 113,000 women. New Engl J Med. 2021;384:428–39. 10.1056/NEJMoa1913948. 33471991 10.1056/NEJMoa1913948 PMC7611105

[6] Kuchenbaecker KB, Hopper JL, Barnes DR, Phillips KA, Mooij TM, Roos-Blom MJ, et al. Risks of breast, ovarian, and contralateral breast cancer for BRCA1 and BRCA2 mutation carriers. J Am Med Assoc. 2017;317:2402–16. 10.1001/jama.2017.7112. 10.1001/jama.2017.7112 28632866

[7] Hu C, Hart SN, Gnanaolivu R, Huang H, Lee KY, Na J, et al. A population-based study of genes previously implicated in breast cancer. New Engl J Med. 2021;384:440–51. 10.1056/NEJMoa2005936. 33471974 10.1056/NEJMoa2005936 PMC8127622

[8] Dowty JG, Win AK, Buchanan DD, Lindor NM, Macrae FA, Clendenning M, et al. Cancer risks for MLH1 and MSH2 mutation carriers. Hum Mutat. 2013;34:490–7. 10.1002/humu.22262. 23255516 10.1002/humu.22262 PMC3887142

[9] Yang X, Mooij TM, Leslie G, Ficorella L, Andrieu N, Kast K, et al. Validation of the BOADICEA model in a prospective cohort of BRCA1/2 pathogenic variant carriers. J Med Genet. 2024. 10.1136/jmg-2024-109943. 10.1136/jmg-2024-109943 PMC11287562 38834293

[10] Lee A, Yang X, Tyrer J, Gentry-Maharaj A, Ryan A, Mavaddat N, et al. Comprehensive epithelial tubo-ovarian cancer risk prediction model incorporating genetic and epidemiological risk factors. J Med Genet. 2022;59:632–43. 10.1136/jmedgenet-2021-107904. 34844974 10.1136/jmedgenet-2021-107904 PMC9252860

[11] Lorenzo F, Xin Y, Douglas FE, Antonis CA. BOADICEA model: updates to the *BRCA2* breast cancer risks for ages 60 years and older. medRxiv [Preprint]. 2024. Available from: 10.1101/2024.04.18.24305970. 10.1038/s44276-024-00079-1 PMC11269170 39072245

